# Prospective evaluation of cerebrospinal fluid levels of β-Endorphin as a predictor of opioid use after scheduled cesarean delivery

**DOI:** 10.21203/rs.3.rs-3125641/v1

**Published:** 2023-07-13

**Authors:** Amelie Pham, Sarah S Osmundson, Alex Pedowitz, Nancy Wickersham, Laura L Sorabella, Stephen Bruehl

**Affiliations:** Vanderbilt University Medical Center; Vanderbilt University Medical Center; University of Miami Leonard M. Miller School of Medicine; Vanderbilt University Medical Center; Vanderbilt University Medical Center; Vanderbilt University Medical Center

**Keywords:** Beta-Endorphin, cerebrospinal fluid, cesarean delivery, endogenous opioid, postoperative opioid use

## Abstract

**Background:**

Prior laboratory work indicates that lower endogenous opioid function is associated with greater analgesic and subjective responses to opioid analgesics. We evaluated whether lower preoperative cerebrospinal uid (CSF) levels of the analgesic endogenous opioid β-Endorphin (BE) were associated with increased opioid use after cesarean delivery (CD).

**Methods:**

We enrolled 136 pregnant women without opioid use or chronic pain who were undergoing CD under regional anesthesia. Preoperatively, participants completed validated pain measures and biospecimens were collected to assess BE levels in plasma and CSF. Postoperatively, pain measures at 48 hours and 2 weeks postpartum were assessed. We evaluated the association between CSF BE levels and total opioid use (in morphine milligram equivalents; MMEs) using linear regression controlling for confounding factors (primary analysis). In secondary analyses, we examined: 1) associations between plasma BE levels and total opioid use, and 2) associations between CSF and plasma BE levels and secondary outcomes (inpatient versus outpatient opioid use, pain intensity).

**Results:**

Participants completed surveys with 100% response rate. The majority were non-Hispanic white (65%), college educated (58%), had private insurance (71%), and had a prior cesarean delivery (69%). Psychiatric diagnoses (depression or anxiety) were common, both currently (22%) and in the past (26%).The median total opioid use across the inpatient and 2-week postpartum follow-up period was 89.1 milligram morphine equivalents (IQR 25–138). Preoperative cerebrospinal uid β-Endorphin levels were not associated with total opioid use (beta = −0.05, SE 0.45, p = 0.64). Similar findings were noted for plasma β-Endorphin levels. cerebrospinal uid β-Endorphin levels were only weakly correlated with plasma β-Endorphin levels (r = 0.30, p < .01). Preoperative cerebrospinal uid and plasma β-Endorphin levels were both positively associated with postpartum pain measures (cerebrospinal uid: at 48 hours, beta = 0.19, SE 0.16, p < 0.05; Plasma: at 48-hours, beta = 0.02, SE 0.03, p = 0.02, and at 2-weeks, beta = 0.27, SE 0.03, p < 0.01).

**Conclusions:**

Lower preoperative cerebrospinal uid levels of β-Endorphin are not associated with increased opioid analgesic use after scheduled cesarean delivery. It is possible that unassessed variability in baseline opioid receptor sensitivity may have confounded ability to test associations between β-Endorphin levels and opioid use outcomes.

## BACKGROUND

Cesarean delivery is the most common major abdominal surgery performed worldwide.^1, 2^ The unique experience of childbirth has unpredictable analgesic requirements, ranging from “none” to “very high”.^3^ Numerous studies have found associations between intense acute postoperative pain and development of chronic pain secondary to nociplastic changes in the central nervous system that enhance pain perception.^4, 5^ Up to 15% of patients suffer from persistent chronic pain after cesarean delivery,^3^ with 4% reporting severe disabling chronic pain,^6^ which can harm a patient’s ability to care for their child, both in the short and long term, by reducing the ability to effectively bond and breastfeed.^7^ Meanwhile, the United States’ opioid epidemic highlights how non-individualized postoperative opioid prescribing has contributed to opioid misuse, diversion, and opioid use disorders.^8^ Approximately 75% of prescribed opioids go unused and 78% of leftover opioids are not disposed of after cesarean delivery, providing a reservoir for potential diversion.^9, 10^ At the same time, a subset of patients use all opioids and report dissatisfaction with postoperative pain control.^9, 11^ Personalized opioid prescribing is therefore at the forefront of contemporary medicine with the goals of optimizing postpartum pain control, preventing chronic pain, and reducing prescribing-related opioid complications. Identifying biomarkers that predict greater analgesic need after surgery could allow for greater individualization of analgesic medications especially opioid analgesics.

The most common opioid medications (e.g. oxycodone) provide pain relief by binding to mu opioid receptors.^12^ Biomarkers re ecting an individual’s opioid system function are hypothesized to predict opioid responsiveness.^12^ Prior laboratory work indexing endogenous opioid system function using opioid blockade methodology comparing pain responses between placebo and naloxone conditions, an opioid antagonist, found consistent associations between lower endogenous opioid function and greater analgesic and risk-relevant subjective responses (e.g., euphoria) to morphine.^13, 14, 15^ These findings suggest that morphine provides less pain relief for those in whom opioid receptor occupancy, by endogenous opioids, is already high.^16^ β-Endorphin (BE) is an endogenous agonist of the mu opioid receptor with analgesic properties similar to morphine. Primary analgesic effects of BE occur in the central nervous system, therefore, BE in the cerebrospinal uid (CSF) is most relevant to analgesia.^12^ Our study evaluated the hypothesis that lower preoperative levels of BE in the CSF are associated with greater opioid use after cesarean delivery.

## METHODS

### Study population

We conducted a prospective cohort study between June 2021 and April 2022 at a single academic center (Vanderbilt University Medical Center; VUMC). Our pool of potential participants were women aged 18 and older undergoing scheduled cesarean delivery under regional anesthesia. We excluded participants receiving general anesthesia, presenting in labor or with unplanned cesarean delivery, or with a prior diagnosis of chronic pain, opioid use disorder, or currently using methadone or buprenorphine. The VUMC Institutional Review Committee approved all study procedures.

### Study protocol and specimen collection

Participants were recruited in the outpatient clinic setting or upon inpatient admission for their scheduled cesarean delivery, during preoperative assessment. All participants provided informed consent. Subsequently, two 4mL samples of maternal blood were collected preoperatively in EDTA tubes for plasma BE assays at the time of routine intravenous (IV) catheter insertion. Upon collection, plasma samples were immediately processed by centrifuge and the supernatant plasma was extracted and stored at −80 degrees Celsius. At the time of regional spinal or combined spinal-epidural placement, a sample of maternal CSF (~ 3.5–4.0mL) was collected by the anesthesiologist prior to administration of any analgesic medication. CSF samples were collected and checked for blood contamination, then centrifuged to discard cellular debris. The supernatant was extracted and stored at −80 degrees Celsius.

At discharge, all participants were provided with 30 tablets of ibuprofen 600mg and 30 tablets of hydrocodone 5mg-acetaminophen 325 mg. Relevant surgical and clinical information was extracted from the electronic health record. Total inpatient opioid use in milligram morphine equivalents (MMEs) was extracted from the electronic medication administration record.

### Pain-related measures

Pain status was assessed via REDCap survey preoperatively (Survey 1) and then again at 48 hours (Survey 2) and 2 weeks (Survey 3) postoperatively. Pain-related measures included: 1) 0–10 numeric rating scale (NRS) measures of average past week and current pain intensity,^17^ 3) the Short Form McGill Pain Questionnaire-2 (MPQ-2),^18^ and 4) the Pain Interference subscale from the Patient Reported Outcomes Measurement Information System (PROMIS) 29 questionnaire (Survey 3 only).^19^ On Survey 3, participants were additionally asked to count and report the number of unused opioid tablets since discharge (from which opioid amount used was calculated in MMEs) and complete questions about their opioid responses. Prior work in this population indicates that self-reported outpatient opioid use corresponds well with opioid use assessed using electronic pill caps.^20^ For individuals reporting ongoing opioid use at Survey 3 (n = 13), number of opioid tablets remaining was re-assessed at 4 weeks postpartum to capture all post-operative opioid use.

### Laboratory Assays

An enzyme-linked immunosorbent assay (ELISA) using a competitive enzyme inhibition immunoassay technique was used to quantify BE levels in plasma and CSF specimens (antibodies-online.com, No. ABIN6955552). Duplicate assays were carried out according to manufacturer instructions, with reported assay sensitivity of 5.11 pg/mL, detection range of 12.35 pg/mL − 1000 pg/mL, and inter- and intra-assay coefficients of variation (CV%) of < 12% for plasma and < 30% for CSF. In our study, because the values for these specimens were in the low range on the standard curve, with many undetectable samples, the standard was further diluted (0.69 pg/mL–1000pg/mL) to move the samples into the detectable range. After determining that the standard curve was linear at lower concentrations, the specimens were measured undiluted using the adjusted standard curve.

Specimens were analyzed in four batches (#1: internal validation for ELISA kit of samples collected from participants 1 to 10, #2: find half of the participants, #3: second half of the participants, #4: re-runs for quality control). Samples with CV% >30% were re-processed and re-analyzed in batch #4 to reduce errors related to sample preparation or ELISA issues. Final data analysis used the mean of the duplicate samples and excluded participants whose CSF samples had CV% >30%. Participants with CSF BE levels below the lower limit of detection were included in the nal analysis and were assigned a value of 0.69 pg/mL.

### Study Exposure and Outcomes

The primary exposure was preoperative CSF BE levels (continuous measure). The secondary exposure was plasma BE levels (continuous measure). Our primary outcome was total postoperative opioid use in MMEs, summed across inpatient and outpatient periods (up to 30 days postpartum). Secondary outcomes included inpatient and outpatient opioid use in MMEs, examined individually, as well as perioperative pain measures.

### Data Analysis

Data were analyzed using Stata/BE software version 17.0 and IBM SPSS Statistics for Windows, Version 28.0 (Armonk, NY). Descriptive statistics (mean ± SD, median [IQR], or % as appropriate) were used to characterize the sample and study outcomes. Zero-order (unadjusted) correlations between BE levels and outcomes were assessed using Pearson correlation coefficients (*r*). Primary analyses examined the association between preoperative CSF BE levels and our primary combined opioid use outcome using hierarchical linear regression, entering potential demographic confounds in step 1 (maternal age, body mass index (BMI), and maternal race and ethnicity) and BE levels in step 2. Secondary analyses used a similar approach to assess associations between preoperative plasma BE levels and our primary outcome, and between preoperative CSF and plasma BE levels and secondary outcomes. Subgroup analysis was performed in participants with prior cesarean section. BE and opioid use outcomes were square root transformed prior to conducting analyses to normalize skewed distributions. All analyses used the maximum number of cases available and used a 2-sided criterion of p < .05 for statistical signicance.

## RESULTS

### Cohort demographics

We recruited 155 participants for the study. Of these, 136 met eligibility criteria, were enrolled, and completed the study (100% response rate for all pre- and postoperative measures). Sample demographic information is reported in Table 1. Participants as a group were relatively young and healthy, with the majority being non-Hispanic white, college-educated with private health insurance, and had undergone cesarean section previously. A self-reported history of depression or anxiety disorders was relatively common. There was a very low rate of postdural headache (2%) and only 2 of 3 participants required a blood patch postpartum. Postpartum complications were rare.

### BE levels and characteristics of opioid use postpartum

Plasma BE levels were available in all 136 participants and CSF BE levels were available for 113 participants. Missing CSF BE data were due to inability to collect CSF samples (n = 7) or CV% issues despite re-processing (n = 16). The mean plasma BE level was 96.4 pg/mL (SD 42.6, n = 136) and mean CSF BE level was 4.2 pg/mL (SD 6.4, n = 113). Final analysis included 39 participants with CSF BE levels below the lower limit of detection. CSF BE levels were significantly associated with plasma BE levels, although the magnitude of the correlation was relatively small (r = 0.30, p < .01).

**Figure 1:**
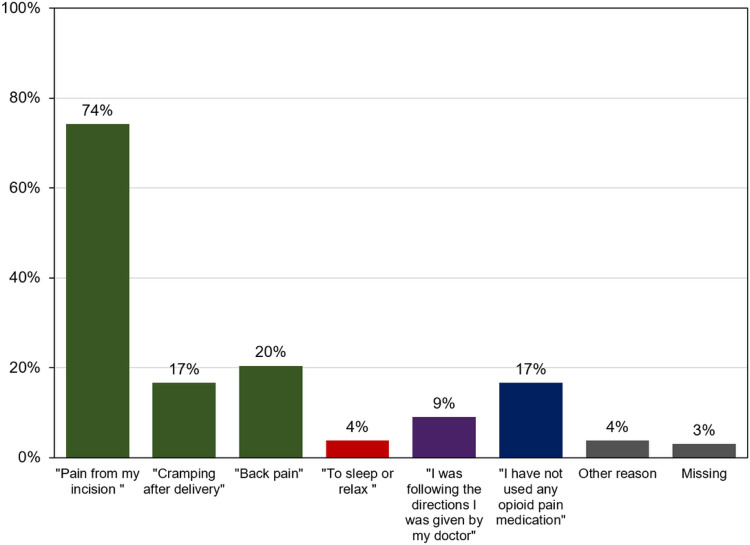
Indications for opioid analgesic medication use after delivery Participants’ responses to the following survey question, “why did you take the opioid analgesic medication you were prescribed from delivery?”

**Figure 2:**
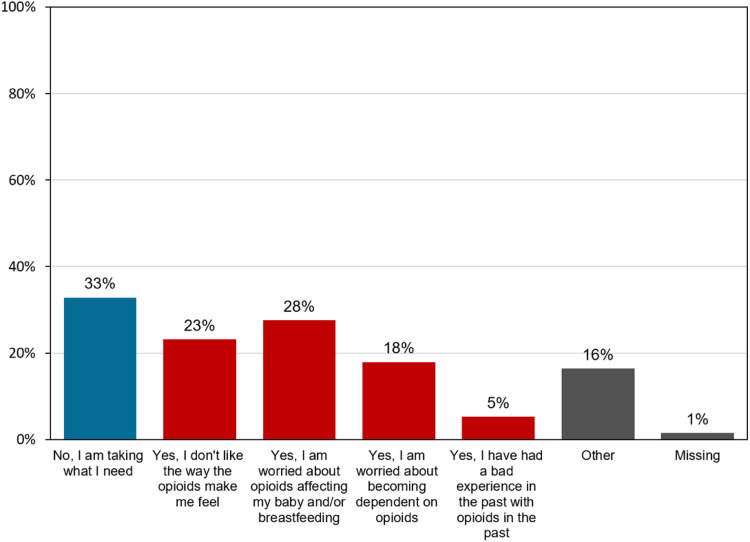
Reasons for limiting use of prescribed opioid analgesic medication after delivery Participants’ responses to the following survey question, “have you tried to limit use of your prescribed opioid pain medication?”

Total median opioid use (inpatient and outpatient periods) was 89.1 MME (IQR 25–138). Total median opioid use during the inpatient admission period was 29.1 MME (IQR 0–41). Speci cally, total median opioid use between 24 and 48 hours postoperatively was 7.5 MME (IQR 0–23). Total median outpatient opioid use was 56.0 MME (IQR 5–100). Participants reported taking prescribed opioid medications for a mean of 5.74 days (SD 4.49). The total median prescribed opioid that went unused in the outpatient setting was 88.0 MME (IQR 40–140). A small proportion of participants additionally received a prescription for alternative non-opioid pain medications prior to discharge postpartum, 5.9% (8/136) for gabapentin and 2.2% (3/136) for cyclobenzaprine. Of the full sample, 25% (34/136) denied any opioid use after discharge and 3% (4/136) took no medications at all for pain after discharge (including acetaminophen and ibuprofen). A small subset of participants (13/136, 10%) reported still taking opioids at 2 weeks postpartum (Survey 3) and 92% (12/13) of these participants had taken all their prescribed opioids on follow-up at 4 weeks postpartum. Only 11 participants (8%) contacted their provider about inadequate pain control after discharge and 4 of them (4/11, 36%) received an additional opioid prescription. Most participants used opioid analgesics for pain management, but a small group (4%) reported using opioids for non-pain related reasons (to sleep or relax) (Fig. 1). Many also reported trying to limit opioid use for various reasons (Fig. 2). Gastroenterological symptoms (62%), such as constipation, nausea, or upset stomach were the most common reported opioid side effects, followed by 30% reporting “feeling out of it”.

### Associations between BE and outcomes

**Figure 3:**
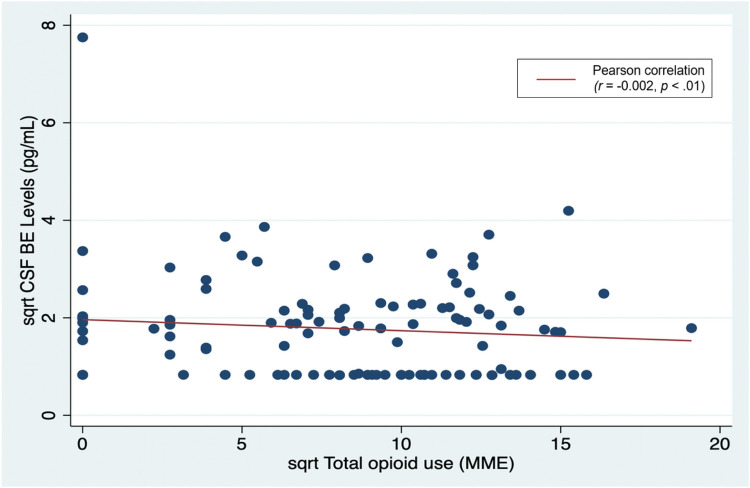
Preoperative CSF β-Endorphin levels and total opioid use after delivery A square root (sqrt) transformation was used to normalize the skewed distribution of preoperative CSF β-Endorphin levels and total opioid use. Total opioid use is calculated from the use summed from both inpatient and outpatient postpartum periods.

Zero-order (unadjusted) correlations between BE levels and all outcomes are summarized in Table 2. Neither CSF nor plasma BE levels were correlated significantly with opioid use outcomes. Plasma BE levels, but not CSF levels, were positively correlated with 2-week postoperative NRS current pain (p < 0.01).Hierarchical linear regressions adjusting for potential confounding in uences of age, BMI, and race indicated that preoperative CSF and plasma levels of BE were not significantly associated with our primary outcome, total opioid use (Table 3 and Fig. 3). Similar analyses indicated that CSF BE was also not signicantly associated with opioid use specically in the inpatient or outpatient settings. Finally, in contrast to negative findings for prediction of opioid use outcomes, hierarchical linear regression analyses indicated that greater CSF BE levels were associated with higher 48-hour postoperative pain intensity on the MPQ-2 (beta = 0.19, SE 0.16, p = 0.049). Associations between plasma BE levels and perioperative pain intensity scores were also signicant for 48-hour and 2-week postpartum NRS current pain intensity ratings (Table 3). Subgroup analysis for participants with a prior cesarean section (n = 94) did not substantively change our findings for our primary and secondary analyses.

## DISCUSSION

Preoperative CSF levels of BE were not associated with opioid analgesic use after scheduled cesarean delivery, even when adjusting for potential confounding covariates. Similarly, no association was found between preoperative plasma levels of BE and opioid outcomes. The sample size available for CSF BE analyses had su cient power to detect a medium effect size (r = 0.31 or greater), with plasma BE analyses able to detect a small effect size (r = 0.24). The study therefore was well-powered to detect associations between BE levels and opioid use, if present, at levels likely to be clinically relevant for inclusion in personalized medicine predictive models. Nevertheless, CSF and plasma BE levels were positively associated with postoperative pain intensity. Intercorrelations between CSF and plasma BE levels were significant but represented only 9% shared variance.

Endogenous opioids, such as BE, activate the same opioid receptors as exogenous opioid medications. Prior studies indicate that lower endogenous opioid system function is associated with greater analgesic and risk-relevant subjective responses when opioids are administered under laboratory conditions.^13, 14, 15^ Operant reinforcement learning models^21, 22^ have led us to hypothesize that individuals, in whom opioids produce more profound analgesia and increased positive subjective effects (e.g., those with low endogenous opioid activity), are more likely to use higher doses of opioids for pain control following cesarean section. However, our results do not support this hypothesis. Because CSF BE levels were assessed only once shortly before delivery, we were unable to test for inverse associations between opioid use and post-surgical CSF BE levels that prior work might predict. Another reason our hypothesis was not supported may relate to how endogenous opioid function was quantied in the current work.Opioid blockade methodology with naloxone used in prior laboratory studies^13, 14, 15^ permitted full capture of opioid system function, that is both endogenous opioid levels (both BE and analgesic enkephalins) and differences in opioid receptor sensitivity. In contrast, CSF BE levels in the current work re ected only BE levels (not enkephalins) and did not address possible in uence of differences in opioid receptor sensitivity. Because use of naloxone for opioid blockade methodology in pregnant women can have potential harmful effects to the fetus, it is di cult to further explore these issues in the cesarean section population. However, use of opioid blockade methodology to assess associations between preoperative endogenous opioid system function and postoperative opioid use in other elective surgery populations may warrant consideration.

Although no association was observed between preoperative BE levels and postoperative opioid use, we did find signicant positive associations between CSF and plasma BE levels and postoperative pain intensity measures. It is important to note that BE derives from two functionally distinct systems in the human body: peripherally (in the plasma) originating primarily from the pituitary gland and centrally (in the CSF) originating from the arcuate nucleus of the hypothalamus and the periaqueductal gray, where the primary analgesic effects of BE occur. Plasma BE is a key modulator of the human stress response^23^ and would be expected to have little analgesic effect. Our findings of positive associations between plasma BE and pain intensity likely reflects links between increased preoperative stress and elevated postoperative pain. Positive associations between preoperative BE in CSF, where analgesic effects are expected, and early postoperative pain intensity is opposite to the predicted direction, potentially reecting pain-related homeostatic demands to increase BE release rather that analgesic effects of that BE release. Meanwhile, the association between plasma and CSF levels of BE has been rarely investigated in humans. Although not a primary aim for our study, our findings of a relatively small but significant correlation between these two BE sources is likely not clinically significant, consistent with the peripheral and central endogenous opioid systems being largely independent.

Whether personalized pain medicine algorithms will ever be clinically feasible remains to be determined.^16^ Individual variability in pain severity after delivery is inuenced by multiple factors.Previously studied risk factors for greater perioperative pain and opioid use in the obstetric population are mostly limited to individual demographics and clinical characteristics,^4, 5, 9, 24, 25, 26, 27, 28, 29, 30, 31^ while the literature in non-obstetric populations suggests that predictors include objective pain responsiveness assessed via quantitative sensory testing, psychosocial characteristics (e.g., depression, pain catastrophizing), and genetic factors.^16^ Though the current results indicate that BE is unlikely to be of value as a biomarker in personalized pain medicine algorithms, alternative biomarkers such as endocannabinoids which have potential interactive effects with endogenous opioids^32, 33^ should be further explored.

Our study provides a template methodology to evaluate other potential predictors of postoperative opioid use that may be more clinically relevant than BE. Given the complexity of the pain experience, predicting postpartum analgesic use will likely require more than a single biomarker. Measures in future studies should be comprehensive and include demographic and clinical information, quantitative sensory testing measures, psychosocial measures, and genetic factors.

Our study has multiple strengths. This is the find prospective study in an obstetric population evaluating a biomarker as a predictor of opioid analgesic use postpartum. We enrolled a large cohort of pregnant patients with a 100% response rate. Standard spinal anesthesia procedures during cesarean section provided us with the opportunity to examine the impact of both peripheral and central endogenous opioid levels on opioid analgesic use outcomes for the find time. We used strict inclusion and exclusion criteria to reduce selection bias. We also used prospective follow up to reduce recall bias. Finally, participants’ electronic medication administration records allowed us to accurately calculate inpatient opioid use and simultaneously evaluate for non-opioid medication use.

This study also had several limitations. We did not assess endogenous opioids prior to pregnancy; whether such measures would be associated with postoperative opioid use is unknown. Furthermore, we only collected biospecimens at one time point so could not evaluate BE biomarker changes after cesarean delivery. Moreover, we only evaluated healthy participants without opioid use disorder, recent opioid use, or chronic pain syndrome, which may have resulted in a narrower range of endogenous opioid levels at baseline. During BE assays, not all samples were analyzed successfully even after re-assay and reasons are unknown. Finally, we relied on self-reported outpatient opioid use. Although evidence suggests these are accurate,^20^ some degree of recall bias is possible.

## CONCLUSION

Preoperative levels of the endogenous opioid BE in the CSF are not associated with opioid analgesic use after scheduled cesarean delivery. Given the clinical importance of optimizing post-surgical pain management while minimizing opioid risks, future studies should investigate other potential patient phenotype predictors of opioid use that may prove useful in a personalized medicine context.

## Figures and Tables

**Table 1:** Demographic and Clinical Characteristics

Variable	Mean ± standard deviation or N (%) (n = 136)
Age (years)	31.0 ± 5.3
Gestational age at delivery (weeks)	38.3 ± 1.5
Gravity	3.3 ± 2.0
Parity	1.5 ± 1.2
Maternal Body Mass Index at delivery (kg/m^2^)	34.2 ± 9.0
Race or ethnicity*	
Non-Hispanic White	89 (65.0)
Non-Hispanic Black	22 (16.1)
Hispanic	14 (10.2)
Asian	4 (2.9)
Unknown or Other	8 (5.8)
Tobacco use*	8 (5.8)
Marijuana use*	7 (5.1)
College education or greater* (n = 131)	76 (58.0)
Prior cesarean	94 (68.6)
Multiple Gestations	3 (2.2)
Public Insurance (n = 129)	37 (28.7)
Obesity (Body Mass Index ≥30 kg/m^2^)	74 (61.7)
Chronic Hypertension	16 (11.8)
Pre-existing diabetes mellitus	8 (5.9)
Psychiatric disorders*	
Current	30 (21.9)
History	36 (26.3)
Currently taking medications	15 (10.9)
Spontaneous or induced labor	5 (3.6)
Hypertensive disorder of pregnancy	14 (10.3)
Length of stay	2.4 ± 0.8
Postdural headache	3 (2.2)
Blood patch required	2 (66.7)

* Self-reported

**Table 2:** Zero-order correlations between β-Endorphin levels and all outcomes

Variables	CSF β-Endorphin levels (n = 113)		Plasma β-Endorphin levels (n = 136)	
	r	p-value	r	p-value
Plasma BE levels	0.300	<0.01*	---	
**Opioid use outcomes**				
Total opioid use	−0.002	0.99	0.101	0.24
Inpatient opioid use	0.009	0.92	0.068	0.43
Outpatient opioid use	0.029	0.76	0.078	0.37
**Perioperative pain measures**				
Preoperative				
NRS Average Pain	0.048	0.64	0.037	0.69
McGill Pain Questionnaire-2	0.074	0.44	0.081	0.35
48-Hour postoperative				
NRS Current Pain	0.158	0.09	0.159	0.07
McGill Pain Questionnaire-2	0.075	0.43	0.086	0.32
2-Week postoperative				
NRS Current Pain	0.077	0.42	0.286	**<0.01** *
McGill Pain Questionnaire-2	0.016	0.87	0.060	0.48
PROMIS Pain Interference	0.041	0.67	0.153	0.08

NRS Average Pain = 0–10 numeric rating scale rating of average pain intensity over the past week,MPQ-2 = McGill Pain Questionnaire Short Form-2 overall pain intensity in the past week, NRS Current Pain = 0–10 numeric rating scale rating of current pain intensity |

* Statistically significant at p < 0.05

**Table 3:** Hierarchical linear regression analyses of preoperative β-Endorphin levels as predictors of primary and secondary outcomes

β-Endorphin Source	R-square change	Beta (± SE)	P-value
Cerebral Spinal Fluid (n = 113)			
Opioid use, Total	<0.01	−0.05 (± 0.45)	0.64
Inpatient	<0.01	−0.03 (± 0.33)	0.79
Outpatient	<0.01	−0.03 (± 0.40)	0.77
Pain Measures			
Preoperative			
NRS Average Pain	0.03	0.19 (± 0.21)	0.06
McGill Pain Questionnaire-2	0.03	0.17 (± 0.14)	0.08
48-Hour			
NRS Current Pain	0.02	0.13 (± 0.08)	0.18
McGill Pain Questionnaire-2	0.04	0.19 (± 0.16)	**<0.05** *
2-Week			
NRS Current Pain	<0.01	0.01 (± 0.07)	0.93
PROMIS Pain Interference	<0.01	−0.05 (± 0.70)	0.61
Plasma (n = 136)			
Opioid use, Total	<0.01	0.07 (± 0.19)	0.40
Inpatient	<0.01	0.08 (± 0.14)	0.36
Outpatient	<0.01	0.05 (± 0.17)	0.60
Pain Measures			
Preoperative			
NRS Average Pain	<0.01	−0.02 (± 0.10)	0.86
McGill Pain Questionnaire-2	<0.01	0.06 (± 0.06)	0.52
48-Hour			
NRS Current Pain	0.04	0.20 (± 0.03)	**0.02** *
McGill Pain Questionnaire-2	0.01	0.08 (± 0.07))	0.36
2-Week			
NRS Current Pain	0.07	0.27 (± 0.03)	**< 0.01** *
PROMIS Pain Interference	0.03	0.16 (± 0.31)	0.06

NRS Average Pain = 0–10 numeric rating scale rating of pain intensity over the past week, MPQ-2 = McGill Pain Questionnaire Short Form-2 overall pain intensity in the past week, NRS Current Pain = 0–10 numeric rating scale rating of current pain intensity |

* Statistically significant at p < 0.05

## Data Availability

The datasets generated and/or analyzed during the current study are not publicly available but are available from the corresponding author on reasonable request.

## Abbreviations

BE: β-Endorphin
BMI: Body mass index
CSF: Cerebrospinal uid
CV: Coefficients of variation
ELISA: Enzyme-linked immunosorbent assay
IV: Intravenous catheter
MMEs: Milligram morphine equivalents
MPQ-2: Short Form McGill Pain Questionnaire-2
NRS: Numeric rating scale
PROMIS-29: Pain Interference subscale from the Patient Reported Outcomes Measurement Information System 29
VUMC: Vanderbilt University Medical Center
